# Mutual absorptive interaction of D-glucose and D-galactose with L-Alanine in the intestine of broiler chickens offered a low-protein wheat-based diet

**DOI:** 10.1016/j.psj.2025.106310

**Published:** 2025-12-18

**Authors:** Julia Riedel, Isabel I. Schermuly, Eva-Maria Saliu, Jürgen Zentek, Jörg R. Aschenbach

**Affiliations:** a Institute of Veterinary Physiology, Freie Universität Berlin, Berlin, Germany; b Institute of Animal Nutrition, Freie Universität Berlin, Berlin, Germany

**Keywords:** Broiler, Hexoses, Intestinal absorption, Low-protein diet, Ussing chamber

## Abstract

This study tested the hypothesis that growth retardation in broiler chickens fed low-protein wheat-based diets is linked to interactions between intestinal hexose and amino acid (**AA**) absorption. A total of 54 male Cobb 500 broilers were fed a three-phase low-protein wheat-based diet for ≥28 days (crude protein: -2% points of Cobb 500 recommendations). Apical jejunal uptakes of L-Alanine (**L-Ala**) were measured in Ussing chambers in the presence of 0, 20 or 100 mM luminal hexoses (D-glucose, D-galactose, D-fructose or D-mannose) and, conversely, D-glucose uptakes were measured in the presence of 0, 20 or 100 mM luminal L-Ala, each in the presence and absence of luminal Na^+^. L-Alanine uptakes were further measured after two preincubation lengths (3 min versus 30 min) with D-glucose or D-galactose and three transepithelial potential differences (***PD*_t_**; open circuit, *PD*_t_ = 0 mV or *PD*_t_ = -50 mV) co-applied with 100 mM luminal D-galactose. With the exception of a statistical trend for L-Ala uptake in the copresence of D-mannose (*P* = 0.087), apical uptakes of L-Ala and D-glucose were generally higher in the presence vs. absence of Na^+^ (*P* < 0.05). Apical L-Ala uptakes showed no sodium × hexose concentration interaction with D-fructose and D-mannose. However, such interaction existed for luminal D-glucose and D-galactose (*P* < 0.05) where D-glucose or D-galactose decreased L-Ala uptakes solely in the presence of Na^+^ (*P* < 0.05). Vice versa, increasing luminal L-Ala concentrations decreased apical D-glucose uptakes solely in Na^+^-containing luminal solutions (sodium × Ala concentration, *P* = 0.011). Different preincubation lengths with hexoses had no interaction effect. However, a *PD*_t_ × sodium interaction (*P* = 0.013) revealed that the Na^+^-dependent portion of L-Ala uptakes was significantly reduced after a 3-min preincubation with 100 mM d-galactose at only -50 mV. The results demonstrate a bidirectional interaction of AA and glucose absorption in the broilers’ jejunum that might be based on subapical sodium accumulation and may at least partially explain reduced growth performance in broilers fed low-protein wheat-based diets.

## Introduction

Feeding low-protein diets to poultry lowers nitrogen (**N**) excretion and production costs (Donato et al., 2016; Onsongo et al., 2018). While many studies reported no inferiority of low-protein diets (Kumar et al., 2016; Shao et al., 2018; Abou-Elkhair et al., 2020), some investigators observed an impaired growth in broiler chickens, particularly when simultaneously replacing corn with wheat as starch source (Chrystal et al., 2021, Greenhalgh et al., 2022). A possible explanation for this phenomenon could be that wheat starch is digested faster than corn starch (Giuberti et al., 2012), resulting in asynchronization of protein and starch digestion dynamics with consequences for glucose and amino acid absorption (Liu and Selle, 2015).

The intestinal absorption of glucose relies on the high affinity Na^+^/glucose cotransporter-1 (**SGLT1**; SLC5A1), which mediates the absorption of D-glucose and D-galactose together with two Na^+^ molecules (Gal-Garber et al., 2000). A Na^+^-dependent high-affinity absorption mechanism was also reported for D-mannose in isolated enterocytes of the chicken small intestine, which showed similar characteristics to the Na^+^-dependent transporter SGLT4 (SLC5A9) in humans (Cano et al., 2001). In contrast, D-fructose absorption occurs independently of Na^+^ through facilitated diffusion via GLUT5 (SLC2A5) (Garriga et al., 2004). Amino acids like L-Ala are absorbed via transporters with overlapping substrate affinities that are either Na^+^-dependent like B^0^AT1 (SLC6A19) and ATB^0,+^ (SLC6A14) or Na^+^-independent like b^0,+^/rBAT (SLC7A9/SLC3A1) (Torras-Llort et al., 2001; Hatanaka et al., 2002; Böhmer et al., 2005).

Given the Na^+^-dependence of SGLT1 and several important AA transporters, it was speculated previously that competition between glucose and AA for the electrochemical Na^+^ gradient might explain lower AA absorption, lower protein accretion and, subsequently, lower growth when easily digestible starch is increased in low-protein diets (Moss et al., 2018). Consequently, we hypothesized that high concentrations of luminal hexoses might inhibit the uptake of AA and, vice versa, high concentrations of luminal AA might inhibit the uptake of glucose in the small intestine of broilers. We further hypothesized that the depressive action of glucose on AA absorption is not linked to the epithelial metabolism of glucose but linked to either the electrical gradient (i.e., membrane potential) and/or the chemical Na^+^ gradient required für Na^+^-dependent transport.

To test these hypotheses, L-Ala was chosen as a model AA because it is a quantitatively significant AA that utilizes different absorption pathways, including several Na^+^-dependent mechanisms (Brady et al., 1979), and because of its limiting role for protein accretion and growth in slow-growing chickens (Niknafs et al., 2022). The different luminal hexoses were selected to investigate sugars that are or are not co-transported with Na^+^ and that are metabolized to a different extent in intestinal epithelial cells.

The resulting objectives of the study were (1) to elucidate a possible inhibition of L-Ala absorption in the copresence of different concentrations of luminal hexoses and, vice versa, the possible inhibition of D-glucose absorption in the copresence of increasing luminal L-Ala concentrations in the jejunum (**JEJ**) of broiler chickens raised on a low-protein wheat-based diet, (2) to examine how the preincubation time with hexoses affects L-Ala uptake in order to evaluate a possible role of intracellular hexose metabolism and (3) to assess the influence of the membrane potential on L-Ala uptake in the presence of high luminal D-galactose to determine whether the electrical gradient or the chemical Na^+^ gradient across the epithelium may be more important for the suspected inhibition of AA absorption by luminal hexoses.

Providing proof for an inhibition of AA absorption by excess glucose and elucidating the mechanisms behind it would greatly advance the understanding of compromised growth performance of broiler chickens receiving low-protein wheat-based diets and might have a great impact on the future formulation of broiler diets.

## Materials and methods

The animal experiments were communicated with the local authority, the State Office for Health and Social Affairs (Reg. No: T 0033/19).

### Animals and diets

In three experimental runs, a total of 54 male Cobb 500 broilers were purchased as day-old chicken from Cobb Germany Avimex GmbH, Wiedemar, Germany. They were floor-housed in the Institute of Animal Nutrition, Freie Universität Berlin, Germany on chopped *Miscanthus giganteus*. The temperature in the barn was kept at 35°C for the first two days and then decreased gradually to 23°C. Artificial light was provided for 24 h for the first two days, for 20 h on the third day and for 18 h per day thereafter. The animals received a three-phase low-protein wheat-based diet (CP, −2% points of Cobb 500 recommendations), that was provided *ad libitum*. The composition of all diets and the analyzed nutrient concentrations can be found in Table 1, Table 2, respectively. The levels of essential AA l-lysine, l-methionine, l-threonine and L-tryptophan met or exceeded the recommendations of Cobb-Vantress in all three diet phases (Cobb&Vantress 2022).

**Table 1:** Feed ingredients and calculated nutrient composition (%, as fed basis).

Ingredients	Starter	Grower	Finisher
Wheat	63.6	69.4	78.1
Soybean meal 48% CP	15.0	11.0	5.00
Rapeseed meal	12.0	9.60	6.00
Mineral premix^1^	1.20	1.24	1.30
Limestone (CaCO_3_)	1.60	1.44	1.20
Soybean oil	4.40	4.83	5.48
Monocalcium phosphate	1.31	1.33	1.36
L-Lysine-HCl^2^	0.48	0.57	0.70
DL-Methionine^3^	0.23	0.30	0.40
L-Threonine^2^	0.17	0.26	0.40
L-Tryptophan^3^	0.01	0.03	0.06

	Calculated nutrient composition		
Dry matter	89.2	86.8	85.3
Crude protein	20.2	17.1	15.1
Crude fiber	3.4	2.9	2.5
Ash	5.08	4.42	3.97
ME (MJ/kg)	12.2	12.6	12.9
Crude fat	6.10	6.73	7.15
Calcium	1.00	0.87	0.78
Phosphorus	0.85	0.76	0.71
SID^3^ Lysine	1.28	1.17	1.09
SID Methionine	0.51	0.58	0.62
SID Methionine + Cysteine	0.91	0.92	0.93
SID Threonine	0.82	0.83	0.84
SID Tryptophan	0.21	0.19	0.18

1 Containing per kilogram premix: 600.000 I.U. vitamin. A (acetate); 120.000 I.U. vitamin D_3_; 8.000 mg vitamin E (a-tocopherol acetate); 200 mg vitamin K_3_ (MSB); 250 mg vitamin B_1_ (mononitrate); 420 mg vitamin B_2_ (crystalline; Riboflavin); 300 mg vitamin B_6_ (pyridoxin-HCl); 1500 mg vitamin B_12;_ 3000 mg niacin (niacinamide); 12500 mg biotin (commercial. feed grade); 100 mg folic acid (crystalline. commercial. feed grade); 1000 mg pantothenic acid (Ca D-pantothenate). 60000 mg choline (chloride); 5000 mg iron (iron carbonate); 5000 mg zinc (zinc sulfate); 6000 mg manganese (manganous oxide); 1000 mg copper (copper oxide); 45 mg iodine (calcium-iodate); 20 mg selenium (sodium-selenite); 140 g sodium (NaCl); 55 g magnesium (magnesium sulfate); carrier: calcium carbonate (calcium min 38%).

2 Ajinomoto/Eurolysine. Amiens. France.

3 SID = standardized ileal digestibility.

**Table 2:** Analyzed values of experimental diets (%, as fed basis).

Nutrient composition	Starter	Grower	Finisher
Dry matter	89.7	89.5	89.7
Crude protein	20.1	16.9	15.9
Crude fiber	3.90	3.26	2.88
Crude fat	6.09	5.51	6.62
Ash	5.73	4.96	4.49
Calcium	1.06	0.86	0.78
Phosphorus	0.71	0.64	0.62
Alanine	0.77	0.62	0.57
Arginine	1.09	0.84	0.78
Asparagine	0.49	0.37	0.34
Cysteine	0.38	0.35	0.34
Glutamic acid	4.21	3.75	3.58
Glycine	0.79	0.65	0.61
Histidine	0.46	0.39	0.35
Isoleucine	0.79	0.63	0.58
Leucine	1.37	1.15	1.06
Lysine	1.31	1.16	1.06
Methionine	0.53	0.55	0.55
Methionine + Cysteine	0.91	0.90	0.90
Phenylalanine	0.87	0.73	0.67
Proline	1.34	1.20	1.17
Serine	0.89	0.74	0.69
Threonine	0.85	0.82	0.79
Tyrosine	0.31	0.50	0.42
Valine	0.90	0.73	0.69

After reaching four weeks of age, two to four chickens per day were randomly selected, stunned and subsequently killed by blood withdrawal. The JEJ was obtained and used for Ussing chamber experiments as described previously (Riedel et al., 2025). The serosal layer of the JEJ was stripped mechanically and the tissue samples were transported to the laboratory in ice-cold buffered solution (Table 3), which was constantly gassed with carbogen (95% O_2_ and 5% CO_2_). Enrofloxacin (final concentration, 0.028 mM) was added to the transport solution to prevent bacterial growth.

**Table 3:** Composition of buffered incubation solutions for Ussing chamber experiments.

Substance	Transport^1^/Serosal	Mucosal + Na^+^	Mucosal Ø Na
	*c_n_ mmol·L^-^*1	*c_n_ mmol·L^-^*1	*c_n_ mmol·L^-^*1
NaCl	85.0	85.0	0.0
NMDG^2^-Cl	0.0	0.0	90.4
NaHCO_3_	25.0	25.0	0.0
Cholin-HCO_3_	0.0	0.0	25.0
KCl	5.40	5.40	0.00
NaH_2_PO_4_	0.60	0.60	0.00
Na_2_HPO_4_	2.40	2.40	0.00
KH_2_HPO_4_	0.00	0.00	0.60
K_2_HPO_4_	0.00	0.00	2.40
HEPES (free acid)	10.0	10.0	10.0
Glucose^3^	20.0	0.0	0.0
D-Mannitol^3^	80.0	0.0	0.0
MgCl_2_	1.20	1.20	1.20
CaCl_2_	3.00	3.00	3.00
Amino acid mix^4^	1.40	no	no

1 Enrofloxacin (final concentration. 0.028 mM) was added to the transport solution to prevent bacterial growth.

2 NMDG = N-methyl-D-glucamine.

3 The mucosal solutions were prepared without 100 mM of hexoses (D-glucose, D-galactose, D-fructose, D-mannose). L-Ala or D-mannitol (as osmotic replacement). The latter additions were made directly in the Ussing chamber.

4 Composition of the amino acid mix can be found in Romanet et al. (2020).

### Feed analysis

The feed analysis followed the methods of the Verband Deutscher Landwirtschaftlicher Untersuchungs- und Forschungsanstalten e.V. (VDLUFA (1976)). Samples were milled (0.5 mm, ZM 200, Retsch GmbH, Haan, Germany) and analyzed for dry matter (**DM**), crude ash, crude protein, ether extract, and AA (Vario Max CN V7.3.9, Elementar Analysesysteme GmbH, Hanau, Germany). Amino acid analysis was performed after HCl hydrolysis by ion exchange chromatography (Amino acid analyzer Biochrom 30, Laborservice Onken GmbH, Gründau, Germany). Calcium was determined via atomic absorption spectrophotometry after dry ashing (Atomic absorption spectrometer contrAA 800, Analytik Jena, Jena, Germany), phosphorus was analyzed photometrically by the vanadate-molybdate method.

### Ussing chamber experiments

The stripped JEJ mucosa samples were cut into squares (∼2 cm^2^) and mounted into conventional Ussing chambers (solution-exposed area, 0.95 cm^2^). The mucosal and serosal sides of the Ussing chambers contained 12 ml of buffered solution that were gassed with carbogen over the whole experimental period. The temperature in the Ussing chamber was maintained at 37°C by a thermostat. The composition of the bathing solutions on the mucosal and serosal sides of the Ussing chamber can be found in Table 3. The mucosal solutions were prepared without hexoses and AA and adjusted to 220 ± 10 mosmol/L. The respective hexoses (D-glucose, D-galactose, D-fructose, D-mannose), L-Ala and/or D-mannitol (as osmotic replacement) were added at the indicated concentration to the mucosal solution reservoir of the individual Ussing chamber immediately before immersing the solution onto the epithelium; thus, the final osmolarity of all solutions was 320 ± 10 mosmol/L. Serosal incubation solutions contained an AA mix consisting of a total of 22 AA (for composition, see Romanet et al. (2020)). All solutions had a pH of 7.40 ± 0.03. Glucagon-like peptide-1 (**GLP-1**) was added to the serosal side of the Ussing chamber (final concentration, 25 nmol/L) to prolong tissue vitality. Bovine serum albumin (final concentration, 100 mg/L) was added to mucosal and serosal solutions to prevent unspecific binding to the equipment and Sab Simplex (final concentration per chamber, 7.0 mg/L) was added to both sides to avoid excessive foaming. All uptake experiments were performed in the presence or absence of mucosal Na^+^. N-Methyl-D-glucamine (**NMDG^+^**) and choline served as Na^+^ replacements in the incubation solutions (Table 3).

Initially all chambers were filled with the appropriate Na^+^-containing solution and tissue samples were allowed to equilibrate for 10 min. The preincubation time of the tissue with luminal hexoses or AA differed for each experimental setup, which will be detailed later in this section. The uptake protocol started by isolating the serosal solution reservoir from the incubation chamber with surgical forceps to eliminate the hydrostatic pressure on the serosal side of the epithelium. Thereafter, the mucosal bathing solution was completely released, and the mucosal solution reservoir was also isolated from the incubation chamber with surgical forceps. The respective Na^+^ or NMDG^+^solution was filled into the mucosal reservoir. Hexoses, AA and/or mannitol were then added to the same reservoir to allow mixing before immersing the solution in the chamber by releasing the surgical forceps, first on the mucosal and then on the serosal side. After a pre-defined period of pre-incubation of the specific experimental setup, 74 kBq of [^3^H] L-Ala or [^3^H] D-glucose were added to the mucosal side of the Ussing chamber together with unlabeled substance to reach a final mucosal concentration of 100 µM. Approximately 20 s after the addition of the radioisotope-labelled L-Ala or D-glucose, two samples (100 µL each) were taken from the mucosal side of the Ussing chamber. After exactly 1 min, the bathing solutions were released on both sides of the Ussing chamber and the JEJ was rinsed three times with 12 mL of ice-cold NMDG^+^ solution to stop ongoing transport processes. The tissue was then processed to determine substrate uptake across the apical membrane into epithelial cells within 1 min (termed 'apical uptake' hereafter) based on the radioactivity of the epithelial lysate as previously described by Riedel et al. (2025).

The proximal and distal parts of JEJ were alternated between treatments over the course of the experimental series to avoid biases resulting from differences in nutrient transport capacities over the length of the JEJ. Additionally, the time order of treatments (e.g., additions of different hexoses) was rotated between experiments to account for sequence effects. Furthermore, pairs of corresponding treatments with mucosal Na^+^-containing vs. NMDG^+^-containing solutions were always treated in parallel by the same operator with a delay of only 2 min to avoid any time bias when comparing Na^+^-containing with corresponding Na^+^-free conditions.

#### Setup 1 – Inhibition of apical L-Ala uptakes by hexoses

For the first experimental setup, a total of 20 Cobb 500 broilers with 12 Ussing chambers per broiler were used to investigate whether the apical uptakes of [^3^H]-labelled L-Ala can be influenced by the presence of 20 mM or 100 mM of four different hexoses (D-glucose, D-galactose, D-fructose, D-mannose). The respective concentrations were used to mirror intermediate and peak physiological glucose concentrations, respectively, given that glucose concentrations in the jejunum of broiler chickens were reported up to ∼50 - 60 mM (Barfull et al., 2002; Mitchell and Lemme, 2008) and might be even higher considering the fast digestibility and high glycaemic index of wheat-based diets (Giuberti et al., 2012). In chambers adjusted to 20 mM and 0 mM hexose, osmolarity was balanced using 80 mM and 100 mM D-mannitol, respectively. The chambers receiving 0 mM hexoses on the mucosal side served as control. Preincubation of tissues with hexoses was maintained for 10 min under open-circuit conditions and under short-circuit conditions thereafter, i.e., with *PD*_t_ clamped to 0 mV. After 10 min under short-circuit conditions, the uptake protocol was started as described earlier in this section.

#### Setup 2 – Inhibition of apical D-glucose uptakes by L-Ala

In a second experimental setup, 10 Cobb 500 broilers with six chambers per broiler were used to investigate whether apical uptakes of [^3^H] D-glucose can be influenced by the simultaneous presence of increasing mucosal L-Ala concentrations (20 mM, 100 mM). Similar to the first experimental setup, the addition of 100 mM of d-mannitol on the mucosal side served as control (0 mM L-Ala). The clamp protocol was the same as for Setup 1, i.e., open-circuit conditions for 10 min and short-circuit conditions (*PD*_t_ = 0 mV) thereafter, with the uptake protocol starting 10 min after switching to short-circuit conditions.

#### Setup 3 – Effect of preincubation period on mucosal L-Ala uptakes in the presence of hexoses

A third experimental setup was used to compare [^3^H] L-Ala uptakes in the JEJ of 12 Cobb 500 broilers (12 Ussing chambers per broiler) after a short (3 min) or a long preincubation period (30 min) of the tissue with either 100 mM of D-glucose or D-galactose on the mucosal side. Ussing chambers that received 100 mM of D-mannitol mucosally served as 0-mM control chambers. After mounting the tissue into the Ussing chambers, an equilibration period was maintained for 10 min under open-circuit conditions (Ruhnke et al., 2013). Afterwards all chambers were short-circuited. For short preincubation of the tissue with luminal hexoses, the Ussing chambers contained 100 mM of D-mannitol on the mucosal side at the beginning. Three minutes before uptake measurements, the buffer solution on the mucosal side was exchanged to fresh Na^+^ or NMDG^+^ solution with either 100 mM D-mannitol, D-glucose or D-galactose. In contrast, long-preincubation chambers received the respective hexoses or mannitol (control) over a period of 30 min. To ensure comparability of the long-preincubation results with the results from the short-preincubation chambers, the mucosal solution of long-preincubation chambers was mock-exchanged with solution of the same composition 3 min before starting uptake measurements.

#### Setup 4 – Effect of membrane potential on mucosal L-Ala uptakes in the presence of hexoses

The fourth experimental setup was intended to discriminate between the role of the electrical gradient (i.e., membrane potential) vs. the chemical gradient (i.e., Na^+^-concentration gradient) for the observed interaction. Uptakes of [^3^H]-L-Ala of 12 Cobb 500 broiler chickens (12 Ussing chambers per broiler) were compared using three different *PD*_t_ in the presence of 100 mM of either D-mannitol or D-galactose. The *PD*_t_ were open circuit, *PD*_t_ = 0 mV (i.e., short circuit) and *PD*_t_ = -50 mV. A decrease of the *PD*_t_ to a stable value of -50 mV should enforce the driving force for both L-Ala and hexoses by hyperpolarization of the apical membrane but should decrease the Na^+^ gradient driving force for L-Ala because of intracellular (subapical) accumulation of Na^+^ due to a massively enforced Na^+^ influx through SGLT1. After mounting the tissue into the Ussing chambers, all chambers were equilibrated for at least 10 min under open-circuit conditions with mucosal solutions containing 0 mM hexoses (i.e., 100 mM D-mannitol). When changing the mucosal incubation solution to fresh Na^+^ or NMDG^+^ solution with or without 100 mM D-galactose, which was 3 min before starting the uptake measurement, the *PD*_t_ was either maintained at open circuit or switched to 0 mV or -50 mV.

### Statistical analysis

Statistical analysis was performed using Sigma Plot 15.0 (Systat Software GmbH, Erkrath, Germany). The data was analyzed using two-way repeated measures (**RM**) ANOVA comparing the factors 'sodium' (Na^+^, NMDG^+^) and 'hexose concentration' (0 mM, 20 mM, 100 mM) in Setup 1 and 'sodium' and 'L-Ala concentration’ (0 mM, 20 mM, 100 mM) for Setup 2. Data of Setup 3 was analyzed by comparing the factors 'preincubation' (short vs. long) and 'sodium' (Na^+^ vs. NMDG^+^) for each of the hexoses. Finally, the factors '*PD*_t_' (open circuit, 0 mV, -50 mV) and 'sodium' (Na^+^ vs. NMDG^+^) were compared for Setup 4. A Student-Newman-Keuls' test was used for post-hoc pairwise comparison. A Shapiro-Wilk test was used to evaluate normal distribution of data sets. In case of missing normal distribution, data was square-rooted before analysis and back-transformed thereafter. Results are given as least square means ± 95% CI. Differences of *P* < 0.05 were considered significant, 0.05 ≤ *P* < 0.10 was considered a trend. The number of experimental animals used is given as n.

## Results

### Setup 1 – Inhibition of apical L-Ala uptakes by hexoses

The results of L-Ala uptakes in the presence of increasing luminal hexose concentrations are presented in Table 4. Uptakes in the presence (mediated by both Na^+^-independent and Na^+^-dependent transport pathways) and in the absence of Na^+^ (mediated by Na^+^-independent transport pathways only) are listed in rows one below the other. The difference between corresponding uptakes in the presence vs. absence of Na^+^ thus represents the uptake via Na^+^-dependent transport pathways.

**Table 4:** Apical uptake of 100 µM L-alanine or 100 µM D-glucose in nmol · cm^-2^ · min^-1^ at increasing luminal concentrations of competing hexoses and L-alanine, respectively in the presence vs. absence of mucosal Na^+^.

Uptake substrate	L-Alanine	L-Alanine	L-Alanine	L-Alanine	D-Glucose
Competing substrate	D-Glucose	D-Galactose	D-Fructose	D-Mannose	L-Alanine
0 mM Competing substrate					
Na^+^	1.47 ± 0.^19A^*	1.35 ± 0.11^A^*	1.68 ± 0.50	1.31 ± 0.09	1.74 ± 0.11^A^*
NMDG^+^	0.76 ± 0.19	0.75 ± 0.11	0.94 ± 0.53	0.95 ± 0.09	0.80 ± 0.11^a^
20 mM Competing substrate					
Na^+^	0.89 ± 0.22^B^	0.76 ± 0.11^B^	1.86 ± 0.53	1.43 ± 0.09	1.48 ± 0.12^B^*
NMDG^+^	0.62 ± 0.19	0.62 ± 0.11	1.05 ± 0.42	0.92 ± 0.09	0.54 ± 0.11^b^
100 mM Competing substrate					
Na^+^	0.77 ± 0.19^B^	0.52 ± 0.13^B^	1.67 ± 0.42	1.48 ± 0.09	1.26 ± 0.11^C^*
NMDG^+^	0.74 ± 0.19	0.59 ± 0.11	1.04 ± 0.42	1.07 ± 0.09	0.65 ± 0.11^ab^
*P*-values					
Concentration	*P* = 0.13	*P* < 0.001	*P* = 0.88	*P* = 0.41	*P* < 0.001
Sodium	*P* = 0.006	*P* = 0.040	*P* < 0.001	*P* = 0.087	*P* < 0.001
Concentration × sodium	*P* = 0.008	*P* < 0.001	*P* = 0.93	*P* = 0.30	*P* = 0.011

N-Methyl-D-glucamine (NMDG^+^) was used as a Na^+^ substitute to create Na^+^-free conditions.

Data represent least square means ± 95 % CI from n = 7-10 chickens.

⁎ Asterisks mark significant differences for the factor sodium within one competing substrate concentration at *P* < 0.05.

A-C Capital letters mark significant differences between competing substrate concentrations in the presence of Na^+^ at *P* < 0.05.

a-b Small letters mark significant differences between competing substrate concentrations in the absence of Na^+^ at *P* < 0.05.

Statistical analysis revealed a significant effect of hexose concentration on L-Ala uptakes for D-galactose only (*P* < 0.001). Significant effects of the factor sodium were observed when L-Ala uptakes were measured in the copresence of D-glucose (*P* = 0.006), D-galactose (*P* = 0.040) and D-fructose (*P* < 0.001) and, as a trend, also for the copresence of D-mannose (*P* = 0.087), indicating that a portion of L-Ala uptake was Na^+^-dependent in all four test conditions. However, significant interactions of hexose concentration × sodium were observed only for the luminal copresence of D-glucose (*P* = 0.008) and D-galactose (*P* < 0.001). Dissecting these interactions further with Student-Newman-Keuls' test, it became evident that, L-Ala uptakes were greater in the presence vs. absence of Na^+^ only in control chambers with no added D-glucose (*P* < 0.05) or D-galactose (*P* < 0.05). In the copresence of 20 and 100 mM of either D-glucose or D-galactose, uptakes of L-Ala in the presence of Na^+^ were decreased (*P* < 0.05) and no longer different from the uptakes in absence of Na^+^. On the other hand, the addition of luminal D-glucose or D-galactose had no effect on L-Ala uptakes in the absence of mucosal Na^+^. Taken together, the results of this setup suggested that concentrations ≥ 20 mM of the SGLT1 substrates D-glucose and D-galactose, but not of the GLUT5 or SGLT4 substrates D-fructose and D-mannose, significantly reduced the Na^+^-dependent portion of the apical uptake of L-Ala at 100 µM L-Ala concentration.

### Setup 2 – Inhibition of apical D-glucose uptakes by L-Ala

The results for d-glucose uptakes in the presence of increasing luminal L-Ala concentrations are also presented in Table 4. Statistical analysis revealed significant main effects of the factors sodium and L-Ala concentration (*P* < 0.001, each). Moreover, a significant interaction existed for L-Ala concentration × sodium (*P* = 0.011). When dissecting this interaction, the addition of 20 mM of L-Ala significantly decreased D-glucose uptakes in comparison to the control irrespective of the presence or absence of mucosal Na^+^ (*P* < 0.05, each). The mucosal addition of 100 mM of L-Ala decreased apical uptakes of D-glucose further only in the presence of Na^+^(*P* < 0.05) but not in its absence. Despite the latter finding, D-glucose uptakes were yet greater in the presence than in the absence of mucosal Na^+^ at all three luminal L-Ala concentrations (*P* < 0.05). Together, these results indicate that very high concentrations of L-Ala (100 mM) appear able to partly inhibit the Na^+^-dependent portion of apical D-glucose uptake at a concentration of 100 µM D-glucose.

### Setup 3 – Effect of preincubation period on mucosal L-Ala uptakes in the presence of hexoses

Setups 1 and 2 were performed using relatively long preincubation periods with the interacting substrate of 20 min before starting the uptake measurement. Such long preincubations may induce changes in epithelial function, for example, due to an intensive metabolism of hexoses within the epithelial cells, which may modify transport gradients or the membrane integration of transport proteins. Therefore, the third experimental setup compared a very short co-incubation period of 3 min with an even longer preincubation period of 30 min. L-Alanine uptakes after the two different preincubation lengths with mannitol (control), D-glucose or D-galactose are presented in Table 5. Two-way RM ANOVA revealed an effect of Na^+^ for all three test conditions (*P* < 0.01) but no effect of the different preincubation lengths. Moreover, no two-way interactions for the factor’s 'preincubation' and 'sodium' were observed. These results suggest that there were no statistically verifiable differences between the two preincubation lengths and that, in contrast to Setup 1, no test condition was able to reduce the Na^+^-dependent portion of L-Ala uptake significantly.

**Table 5:** Apical uptake of 100 µM L-alanine in nmol · cm^-2^ · min^-1^ after short (3 min) or long (30 min) mucosal preincubations with 100 mM of either D-mannitol (control), D-glucose or D-galactose in the presence vs. absence of mucosal Na^+^.

Competing substrate	D-Mannitol	D-Glucose	D-Galactose
Short preincubation			
Na^+^	1.00 ± 0.09	0.85 ± 0.08	0.81 ± 0.12
NMDG^+^	0.81 ± 0.09	0.58 ± 0.10	0.53 ± 0.12
Long preincubation			
Na^+^	0.95 ± 0.09	0.99 ± 0.10	0.60 ± 0.12
NMDG^+^	0.78 ± 0.09	0.60 ± 0.08	0.47 ± 0.12
*P*-values			
Preincubation	*P* = 0.28	*P* = 0.33	*P* = 0.24
Sodium	*P* < 0.001	*P* < 0.001	*P* = 0.004
Preincubation × Sodium	*P* = 0.81	*P* = 0.25	*P* = 0.22

N-Methyl-D-glucamine (NMDG^+^) was used as a Na^+^ substitute to create Na^+^-free conditions.

Data represent least square means ± 95 % CI from n = 11-12 broiler chickens.

### Setup 4 - Effect of membrane potential on mucosal L-Ala uptakes in presence of hexoses

Results of the two-way RM ANOVA comparing the factors '*PD*_t_' and 'sodium' can be found in Table 6. In the absence of mucosal hexoses (control), uptakes of L-Ala where strongly Na^+^-dependent (*P* < 0.001) but the *PD*_t_ had no effect, and no two-way interaction was found between potential difference and sodium. In contrast, two-way RM ANOVA of L-Ala uptakes in the presence of 100 mM of luminal D-galactose revealed significant effects of both *PD*_t_ (*P* = 0.047) and sodium (*P* < 0.001). Moreover, *PD*_t_ × sodium interacted significantly (*P* = 0.013). While L-Ala uptakes in the presence of 100 mM D-galactose did not differ under open-circuit and 0-mV conditions, clamping the *PD*_t_ to -50 mV resulted in significantly lower L-Ala uptakes in the presence of Na^+^ (*P* < 0.05). Furthermore, Na^+^-dependent uptakes of L-Ala were only present under open- and 0- mV conditions (*P* < 0.05), while L-Ala uptakes under -50- mV conditions did not differ between Na^+^ and NMDG^+^.

**Table 6:** Apical uptake of 100 µM L-alanine in nmol · cm^-2^ · min^-1^ in the presence of either 100 mM of mucosal D-mannitol (control) or D-galactose at three different transepithelial potential differences (*PD*_t_) in the presence vs. absence of mucosal Na^+^.

Competing substrate	D-Mannitol	D-Galactose
Open circuit		
Na^+^	1.18 ± 0.09	0.97 ± 0.10^A^*
NMDG^+^	0.77 ± 0.09	0.57 ± 0.10
0 mV		
Na^+^	1.01 ± 0.09	0.83 ± 0.10^A^*
NMDG^+^	0.67 ± 0.09	0.53 ± 0.11
-50 mV		
Na^+^	1.16 ± 0.10	0.59 ± 0.11^B^
NMDG^+^	0.63 ± 0.10	0.52 ± 0.10
*P*-values		
*PD*_t_	*P* = 0.57	*P* = 0.047
Sodium	*P* < 0.001	*P* < 0.001
*PD*_t_ × sodium	*P* = 0.63	*P* = 0.013

N-Methyl-D-glucamine (NMDG^+^) was used as a Na^+^ substitute to create Na^+^-free conditions.

Data represent least square means ± 95 % CI from n = 11-12 broiler chickens.

A-B Capital letters mark significant differences between *PD*_t_ in the presence of Na^+^.

⁎ Asterisks mark significant differences between Na^+^ and NMDG^+^within one *PD*_t_.

## Discussion

The present study hypothesized that the frequently observed decrease of growth performance in broiler chickens receiving a low-protein wheat-based diet might result from an interaction between glucose and AA absorption in the small intestine of birds. Therefore, the aim of the study was to investigate the absorption of L-Ala in the presence of different luminal hexose concentrations and, vice versa, the absorption of D-glucose in the presence of different L-Ala concentrations in the JEJ of broiler chickens after pre-feeding a low-protein wheat-based diet. Using the Ussing chamber technique, we further investigated the influence of two different preincubation times with luminal hexoses on L-Ala uptake and determined the influence of three different *PD*_t_ on L-Ala uptake in the JEJ of broilers.

The results of the performed experimental setups consistently revealed Na^+^-dependent uptakes of L-Ala under control conditions, which agrees with the absorption mechanisms reported in literature (Broer, 2008; Wright et al., 2018). In the first experimental setup, a concentration of 20 mM or 100 mM of either luminal D-glucose or luminal D-galactose impeded the active Na^+^-dependent portion of L-Ala uptake at 100 µM L-Ala concentration, whereas D-fructose and D-mannose had no influence. Similar inhibitory effects on the absorption of AA in the presence of hexoses have previously been observed in other animal species and were attributed to the formation of potentially toxic metabolites (Saunders and Isselbacher, 1965), to the competition for the same energy source (Newey and Smyth, 1964) or to allosteric interactions at the outer side of the BBM (Alvarado, 1966). However, the results of the present study suggest that all these previous explanations, which are largely based on older experimental approaches and date back several decades, do probably not apply to the observed interaction between hexoses and AA in the small intestine of broiler chickens.

Given that the small intestinal absorption of D-glucose and D-galactose occurs mainly through the Na^+^-dependent SGLT1 (Wright, 1993) and the apical absorption of the neutral AA L-Ala is also prominently mediated through Na^+^-dependent AA transporters like B^0^AT1 and ATB^0,+^ (Broer, 2008), it is likely that the observed interaction between hexoses and L-Ala in our study results from the common use of the transepithelial Na^+^-gradient. An inhibitory effect of D-galactose on cycloleucine uptake was already reported many years ago and was related to the Na^+^-dependency of both systems in the dogfish’s intestine (Read, 1967). In contrast, Kimmich and Randles (1973) argued against a role of the Na^+^-gradient in isolated intestinal cells of chickens. Strengthening the Na^+^-gradient hypothesis, D-fructose, which is absorbed independently of Na^+^ through facilitative diffusion (Garriga et al., 2004), had no influence on L-Ala uptakes in the present study. Robinson and Alvarado (1971) also reported no effect of D-fructose on L-Ala absorption in the rabbit intestine. Interestingly, increasing luminal concentrations of D-mannose had no influence on L-Ala uptake in our study, although D-mannose was reported to be absorbed in cotransport with Na^+^ in small intestinal epithelial cells of chickens (Cano et al., 2001). This could be attributed to the fact, that D-mannose is co-transported with only one Na^+^ ion (in contrast to the two Na^+^ ions of SGLT1) and that the V_max_ of the mannose transport system is about 1000 times lower than that of SGLT1 (Cano et al., 2001).

In the second experimental setup, the present study revealed strongly Na^+^-dependent uptakes of D-glucose in the JEJ of broiler chickens even in the presence of increasing luminal L-Ala concentrations. Nevertheless, 20 mM and 100 mM of luminal L-Ala impeded D-glucose uptakes in the presence of mucosal Na^+^ but to a lesser extent than the SGLT1 hexoses had impeded L-Ala uptakes in the first experimental setup. In accordance with this, it was reported that the non-metabolizable SGLT-1 substrate 3-O-methyl-D-glucose **(3-OMG**) inhibited 60% of valine uptake, whereas only 20% of 3-OMG accumulation was inhibited by the presence of valine in isolated cells of the chicken intestine (Saunders and Isselbacher, 1965). With regard to the Na^+^-gradient, this could probably be attributed to the fact, that D-glucose and D-galactose are co-transported with two Na^+^ ions by SGLT1 (Wright, 1993), whereas L-Ala is primarily co-transported by B^0^AT1 with only one Na^+^ ion (Böhmer et al., 2005). The lower Na^+^-influx via B^0^AT1 might not disrupt the transepithelial Na^+^-gradient to the same extent as increasing luminal concentrations of D-glucose or D-galactose.

In BBM vesicles of the rat small intestine, Murer et al. (1975) once reported that preincubation with L-Ala for 60 min abolished the inhibitory effect of the AA on D-glucose transfer. Yet, Read (1967) reported a more effective inhibition of cycloleucine uptakes by D-galactose after 10 min compared to 2 min preincubation in the dogfish’s intestine. The third experimental setup therefore investigated the influence of a short (i.e., 3 min) and a long preincubation period (i.e., 30 min) with 100 mM luminal hexose on the apical L-Ala uptake in the JEJ of broiler chickens. Short and long preincubation periods with D-glucose or D-galactose did not differ in their effect on L-Ala uptakes, which suggests that the observed interaction does not result from intracellular hexose metabolism.

At variance to our results in the first experimental setup, neither short nor long preincubation with 100 mM luminal D-glucose or D-galactose impeded the Na^+^-dependent component of L-Ala uptakes in Setup 3. The varying effects of hexoses on apical L-Ala uptake might have resulted from small methodological differences of Setup 3 vs. Setup 1 (e.g., the different time windows or the exchange of incubation solution before uptake). They could also have originated from subtle variations in the viability of tissues used or from variations in animal batches and their 'biological' age. Regarding the latter, it has been shown that active glucose transport across the jejunal epithelium decreases with age in broiler chickens (Shibata et al., 2019, 2023). Importantly, all these explanations are not in contrast to our hypothesis of reciprocal inhibition of Na^+^-dependent transport of hexoses and AA. They can be seen in line with the observation that we still do not understand all the circumstances that determine whether a wheat-based low-protein diet impedes broiler growth or not.

The discussion to this point proceeded from the assumption that Na^+^ accumulation particularly in the subapical space causes a decreased Na^+^ driving force for L-Ala uptake into the cell. Murer et al. (1975) had claimed, that not Na^+^ accumulation but the depolarization of the apical cell membrane would inhibit the electrical driving force for AA entry. The first experimental setup of this report had already questioned the *PD* concept because the results were obtained under short-circuit conditions where membrane *PD* deviations are largely neutralized by an external clamp current. To further substantiate this finding, L-Ala uptakes were tested in the presence of either 100 mM of luminal D-galactose or D-mannitol (control) at three different transepithelial *PD*_t_: Open-circuit conditions would allow depolarization of the membrane by Na^+^ influx and thus maximize a membrane potential-dependent inhibition of L-Ala uptake whereas short-circuit conditions would largely abolish membrane potential effects on L-Ala uptake. On the other hand, clamping the tissue to -50 mV would stably hyperpolarize the apical membrane (inside negative) and thus enhance the electrical driving force for the apical Na^+^-cotransport of both L-Ala and D-galactose. According to this concept, the L-Ala uptake should rise in the presence of D-galactose if the interaction was predominantly based on *PD*_t_ effects. By contrast, clamping to -50 mV would decrease L-Ala uptake if the interaction was primarily dependent on the Na^+^-gradient.

Under control conditions the three *PD*_t_ had no effect on the Na^+^-dependent uptake of L-Ala. In contrast, clamping the *PD*_t_ to -50 mV in the presence of 100 mM D-galactose resulted in a loss of the Na^+^-dependent component of L-Ala uptake. Thus, we conclude that the interaction between D-galactose and L-Ala absorption resides on a subapical intracellular Na^+^ accumulation. According to the accelerated efflux theory, the absorption of sugars and AA increase the intracellular Na^+^ concentration that can stimulate Na^+^-dependent efflux of a substrate on the one hand and inhibit the Na^+^-dependent substrate influx on the other hand (Semenza, 1971).

In conclusion, our study revealed an interaction between the Na^+^-dependent portions of AA and D-glucose and D-galactose absorption in the small intestine of broiler chickens raised on a low-protein wheat-based diet. The bidirectional inhibition of L-Ala uptake by D-glucose and D-galactose on the one hand and of D-glucose uptakes by L-Ala on the other hand was concentration dependent. Notably, the Na^+^-dependent portion of L-Ala uptake could be inhibited almost completely by 20 mM glucose, a concentration that should be exceeded when feeding easily digestible carbohydrate sources like wheat starch. Consequently, an inhibition of AA absorption by high luminal glucose in the proximal intestine could partly explain the growth retardation by easily digestible starch in low-protein diets, as well as the remediating effect of slower digestible starch on protein accretion and growth. It would act jointly with the unfavourable effects of asynchronous provision of glucose and AA on AA deamination rates (Selle et al., 2022) and other postabsorptive metabolic effects that decrease N deposition into muscle protein when feeding rapidly digestible starch (Luo et al., 2025). Mirroring the controverse data on growth performance, however, the inhibitory effect of high luminal hexose concentrations on AA absorption were not consistent over the different experimental setups in the present study. This indicates that we currently do not understand all factors that influence this interaction. The present clamping experiments suggest that a subapical Na^+^ accumulation could at least partially explain the bidirectional inhibition of glucose and AA absorption.

From a practical point of view, future feeding trials should investigate whether growth retardation by wheat-based low-protein diets can be ameliorated by providing more luminal Na^+^, i.e., by increasing the dietary Na^+^ content to the upper range of the accepted safety margins. It should also be noted, that the present study investigated AA and hexose interactions only in birds pre-fed a low-protein wheat-based diet. Future mechanistic studies should include pre-feeding with standard-protein diets, as well as different starch sources, to elucidate whether a certain pre-feeding predisposes to this interaction.

## Declaration of AI and AI-assisted technologies in the writing process

During the preparation of this work no AI-assisted technologies were used.

## Funding

This work was supported by an Elsa Neumann Grant of the State of Berlin to JR [grant 074025]. The publication of this work was supported by Open Access Publishing Funds of Freie Universität Berlin.

## CRediT authorship contribution statement

**Julia Riedel:** Writing – review & editing, Writing – original draft, Project administration, Methodology, Investigation, Formal analysis, Conceptualization. **Isabel I. Schermuly:** Writing – review & editing, Methodology, Investigation, Conceptualization. **Eva-Maria Saliu:** Writing – review & editing, Methodology, Conceptualization. **Jürgen Zentek:** Writing – review & editing, Resources, Methodology. **Jörg R. Aschenbach:** Writing – review & editing, Writing – original draft, Supervision, Resources, Project administration, Methodology, Conceptualization.

## Disclosures

All authors have no conflict of interest to declare.
